# Employment conditions and leisure-time physical activity among Korean workers: a longitudinal study (2009–2019)

**DOI:** 10.1186/s12889-023-15766-w

**Published:** 2023-05-25

**Authors:** Chungah Kim, Hyunju Jin, Gabriel John Dusing

**Affiliations:** 1 School of Kinesiology and Health Science, York University, Toront, Canada; 2 College of Physical Education and Health Sciences, Zhejiang Normal University, Jinhua, China; 3 Seoul National University Institute of Sport Science, Seoul, South Korea

**Keywords:** Leisure-time physical activity, Working condition, Employment condition, Longitudinal study, South Korea

## Abstract

**Background:**

Employment conditions may affect individuals’ leisure-time physical activity (LTPA). We aimed to examine the relationship between changes in working and employment conditions and LTPA among working-age populations in South Korea from 2009 to 2019.

**Methods:**

A cohort of 6,553 men and 5,124 women aged 19–64 years was analyzed using linear individual-level fixed-effects regressions to examine changes in working and employment conditions with changes in LTPA.

**Results:**

Reduced working hours, labor union membership, and part-time work were associated with increased LTPA for both sexes. Manual labor and self-reported precarious work were associated with reduced LTPA. The longitudinal relationship between employment conditions and LTPA was clear in men, but less apparent in women.

**Conclusions:**

Changes in working and employment conditions had longitudinal associations with changes in LTPA among working-age Koreans. Future research should examine changing employment conditions and their effect on LTPA, particularly among women and manual/precarious workers. These results could inform effective planning and interventions to increase LTPA.

**Supplementary Information:**

The online version contains supplementary material available at 10.1186/s12889-023-15766-w.

## Background

Sufficient physical activity is known to reduce the risk of premature mortality by 20‒30% [1]. The 2018 physical activity guidelines for Americans recommend a minimum of 150‒300 min/week of moderate physical activity to lower the risk of all-cause, cardiovascular disease, and non-cardiovascular disease mortality by approximately 19–25% [2]. LTPA attenuates the adverse effects of obesity on non-communicable diseases [3]. More specifically, a large-scale study shows LTPA to be associated with reductions in breast (6‒10% reduction), endometrial (10‒18%), kidney (11‒17%), and liver (18‒27%) cancer risks [4]. Additionally, a Finnish study found that increasing levels of LTPA participation were positively associated with physical health functioning. Specifically, increasing LTPA participation from low to moderate levels of participation had a beta of 3.08, and increasing from low to vigorous had a beta of 4.67 [5].

Working and employment conditions can be facilitators or barriers for LTPA in multiple ways: (1) The characteristics of physical activity on the job may either facilitate or impede workers’ motivations for LTPA. For example, a non-manual worker who mostly sits on the job tends to feel the need for LTPA. In contrast, a manual worker who stands for a long time or carries heavy loads may lose motivation to engage in LTPA due to physical fatigue [6]. (2) Time availability due to the nature of a job may also influence the likelihood of partaking in LTPA. For example, employees who do prolonged physical labor or need to rest during the day after working night shifts may have difficulty finding time to participate in LTPA [7, 8]. (3) Degrees of job control and psychological demands influenced by levels of job security may be related to sedentary lifestyles with low LTPA [9, 10]. A greater level of job control and autonomy may provide individuals with more flexibility in their work schedule and responsibilities, allowing them to better balance work and leisure activities. This could make it easier for individuals to schedule time for LTPA, which may lead to higher levels of participation. For example, a precarious worker with an insecure job may be less likely to spend time on leisure activities, such as LTPA [11].

However, studies examining the association between employment conditions and LTPA are still scarce. Only one cross-sectional study has examined the association between working and employment conditions and LTPA in South Korea. It found that long working hours and self-reported precarious employment, shift, and manual were associated with less LTPA [11]. Internationally, more studies have focused on the relationship between LTPA and various work-related factors, but not employment conditions [10, 12]. Those studies found that while there was evidence suggesting an association between LTPA and whether one was a manual or a non-manual worker, there was no consistent association between full/part-time workers and LTPA levels related to psychosocial work demands and longer or shorter working hours.

Therefore, expanding on previous research on the cross-sectional association between employment conditions and LTPA, this study aimed to examine whether changes in these conditions were associated with changes in LTPA among working-age populations in South Korea. Specifically, this study focused on conditions such as working hours; union membership; having shift, manual, part-time,, or at-home work; having (self-reported) precarious employment conditions; or having fixed-term employment. In addition, we examined whether the association between employment conditions and LTPA differed by gender.

## Methods

### Study population

We obtained data from the Korean Labor & Income Panel Study (KLIPS), a nationally-representative panel survey that follows approximately 5,000 urban households in South Korea [13]. Household respondents over the age of 15 are interviewed annually. At the time of our study, 22 waves were available for analysis (1998‒2019). However, given that a new cohort had been added in 2009, we used waves 12‒22 (2009‒2019). Further information about this panel and the data is publicly available at https://www.kli.re.kr/klips_eng/index.do.

Among 15,810 working-age people (aged 15‒64 in 2009) surveyed during the study periods, the analysis included people who responded that they had been employed for at least two or more years as salaried workers. The baseline cohort included 6,553 (56%) men and 5,124 (44%) women born in 1945 or later. The appendix presents the flowchart of the sample inclusion/exclusion criteria.

### Outcomes

We conducted a study to assess the self-reported time spent on LTPA per session. LTPA was assessed using the question, “*Do you exercise regularly?”* This question was asked to those who chose the *exercise* option in response to the question: “*On a daily basis, what do you do to maintain your health? Please mark three options in the order of frequency.”* The options included: (1) *exercise*; (2) *dietary control*; (3) *reduced consumption of cigarettes or alcohol*; (4) *herbal medicine or nutritional supplements*; (5) *communal bathhouses* (e.g., bath, sauna, steam bath, or similar activities) (6) getting enough rest and sleep; (7) *periodic medical checkups;* (8) *other*; and (9) *nothing*. Participants who chose *exercise* as one of the three options were then asked to choose whether the exercise was: (1) *regular*, (2) *irregular or on a casual basis*, or (3) *almost never* [14]. Participants who reported exercising regularly, irregularly, or almost never were then asked to indicate how often they exercised per month and the duration of each exercise session in hours and minutes. The degree of leisure-time physical activity (LTPA) is typically measured by assessing the type, intensity, and frequency of activities [15], or by determining the metabolic equivalent task (MET) based on the frequency and duration of specific LTPA pursuits [7]. However, the Korean Labor and Income Panel Study (KLIPS) only offers data on frequency and duration, without information on the type or intensity of LTPA. Consequently, we calculated the total number of minutes dedicated to exercise per month and employed this as a continuous scale for our primary outcome.

### Exposures: Working and employment conditions

Working and employment conditions were measured using nine factors, as suggested by the KLIPS guideline [16]: (1) *work hours*: respondents were asked an open-ended question about how many hours they worked a week; (2) *union membership*: (no/don’t know vs. yes); (3) *shift work*: respondents were asked whether they worked in shifts; (4) *manual labor*: defined according to the 2017 Occupation and Industry Codes. A code over 600 indicated a manual worker, (5) *part-time work*, (6) *work at home*, (7) *precarious work*, and (8) *fixed-term employment* (*temporary contract work)*.

### Covariates

Our study used within-person estimators (*individual fixed-effects*), which accounted for observed and unobserved time-invariant confounders (i.e., gender, personality, and childhood experiences). To adjust for time-variant confounders, we additionally included the following variables: log-transformed age (continuous), marital status (*married/unmarried/divorced/separated/widowed*), education (*no schooling, primary*, or *middle school/high school/any post-secondary/bachelor’s/postgraduate/missing*), smoking (*yes/no/used to/missing*), alcohol use (*yes/no/used to/missing*), self-reported health (*very good/good/fair/bad/very bad/missing*), year fixed-effect to control for macroeconomic fluctuations, and the province of residence (17 regions as dummy variables). All covariates were recoded to comply with KLIPS guidelines [16].

### Statistical analysis

To examine changes in working and employment conditions related to LTPA, we conducted fixed-effects regression, which identifies within-group variation over time. A linear model with robust standard errors was used for the multinomial outcome of LTPA because of ease of interpretation with coefficient changes understood as probability changes and it considers all within-person changes of the participants as opposed to a logit model [17]. To address missing values in the covariates, we conducted a complete case analysis by including all the missing values as an incomplete dataset using maximum likelihood estimation. Robust standard errors, clustered at the individual level, were used for the analyses. For each model, gender-stratified analyses were conducted, given the gender difference in LTPA identified in prior literature [11, 18].

We conducted several sensitivity analyses. First, instead of designing linear regression modeling, we conducted logistic fixed-effects regression for a binary outcome (exercise regularly vs. the other options) to ensure that our use of linear regression modeling was not biased, despite the violation of the linear regression assumption [19]. Second, we included a one-year lagged outcome in our main linear and logistic fixed-effect regression models, which assessed the time spent on LTPA per month. By doing this, we aimed to investigate whether there was a temporal association between current levels of LTPA and previous working conditions. This analysis allowed us to explore whether the current levels of physical activity were influenced by past working conditions [20].”

## Results

The baseline descriptive statistics of the participants (n = 11,677) are presented in Table 1. The distribution of our sample showed that 67.15% of the men and 20.65% of the women participated in LTPA. Notably, baseline LTPA activity was higher among males, married persons, those with higher educational attainment, who used to smoke, and engaged in shift, part-time, unionized, or non-manual work. The change in the proportion of those who participated in LTPA over the period covering waves 12‒22 ranged from 23.97 to 28.28% of the sample.

**Table 1:** Baseline characteristics of the study population

Sample characteristics		Proportions% (N = 11,677)	% of LTPA (N = 2968)
Gender	Men	56.12 (6553)	64.35 (1910)
	Women	43.88 (5124)	35.65 (1058)
p-value < 0.001			
Education	Middle school or less	13.39 (1563)	9.03 (268)
	High school	33.12 (3868)	30.15 (895)
	Junior college	18.69 (2183)	17.62 (523)
	Bachelor’s degree or more	34.79 (4062)	43.19 (1282)
	Missing	0.01 (1)	0.00 (0)
p-value < 0.001			
Smoking	Smoker	27.19 (3175)	26.04 (773)
	Smoked in the past	6.75 (788)	10.55 (313)
	Never smoked	66.04 (7712)	63.41 (1882)
	Missing	0.02 (2)	0.00(0)
p-value < 0.001			
Alcohol	Consume alcohol	67.65 (7900)	71.66 (2127)
	Consumed alcohol in the past	1.82 (212)	2.32(69)
	Never consumed alcohol	30.51 (3563)	26.01 (772)
	Missing	0.02 (2)	0.00 (0)
p-value < 0.001			
Self-reported health	Very good	7.27 (849)	8.19 (243)
	Good	62.18 (7261)	67.52 (2004)
	Fair	25.89 (3023)	19.98 (593)
	Bad	4.26 (498)	4.11 (122)
	Very bad	0.36 (42)	0.17 (5)
	Missing	0.03 (4)	0.03 (1)
p-value < 0.001			
Marital status	Unmarried	29.47 (3441)	27.26 (809)
	Married	62.89 (7344)	66.17(1964)
	Separated	0.85 (99)	0.57 (17)
	Divorced	4.14 (483)	3.84 (114)
	Widowed	2.65 (310)	2.16 (64)
	Missing	0.00 (0)	N/A
p-value < 0.001			
Labor union	Yes	14.21 (1659)	20.25 (601)
	No	82.86 (9675)	77.26 (2293)
	Don’t know	2.94 (343)	2.49 (74)
p-value < 0.001			
Shift labor	Yes	9.21 (1076)	11.08 (329)
	No	90.79 (10,601)	88.92 (2639)
p < .001			
Physical labor	No	63.20 (7380)	68.73 (2040)
	Yes	36.80 (4297)	31.27 (928)
p < .001			
Part-time work	Yes	70.17 (8194)	75.64 (2245)
	No	29.83 (3483)	24.36 (723)
p-value < 0.001			
Work at home	Yes	0.92 (108)	1.04 (31)
	No	99.08 (11,569)	98.96 (2937)
p = .112			
Precarious Employment	Yes	8.26 (964)	5.26 (156)
	No	91.74 (10,713)	94.74 (2812)
p-value < 0.001			
Fixed-term Employment	Yes	29.83 (3483)	24.36 (723)
	No	70.17 (8194)	75.64 (2245)
p-value = 0.002			
Age < 25	Yes	11.43 (1335)	13.17 (391)
	No	88.57 (10,342)	86.83 (2577)
p-value < 0.001	< 25	29.10 (3398)	31.50 (935)
Working hours	25‒44	53.15 (6206)	53.64 (1592)
	45‒64	17.75 (2073)	14.86 (441)
p-value < 0.001	< 40 h per week	50.67 (5917)	52.63 (1562)

Note: LTPA, leisure-time physical activity

Table 2 presents the results of individual-level fixed-effects regression models. In Model 1, the unadjusted fixed-effects model for the probabilities of LTPA (an additional table file shows this in more detail [see Additional File 1, Supplementary Table 2]) indicated that changes in working conditions were associated with LTPA.

**Table 2:** Individual level fixed-effects models predicting the effect of change in working and employment conditions on the levels of leisure-time physical activity

Predictors	Model 1: Total (95% CI)	Model 2: Men (95% CI)	Model 3: Women (95% CI)
Working hours	-12.02*** (-19.22, -4.83)	-11.04* (-20.49, -1.59)	-13.58* (-24.45 to -2.71)
Labor union	30.49** (7.76, 53.23)	44.36** (14.82, 73.89)	3.06 (-30.85, 36.98)
Shift work	20.88 (-6.14, 47.90)	34.03 (-3.77, 71.84)	1.42 (-32.44, 35.28)
Manual labor	-42.07** (-71.86, -12.27)	-64.32** (-106.36, -22.29)	-1.32 (-39.74, 37.10)
Part-time work	50.29** (14.98, 85.60)	49.55 (-29.78, 128.87)	41.60* (3.62, 79.58)
Work at home	-8.00 (-77.05, 61.04)	-21.05 (-131.26, 89.16)	-6.04 (-90.43, 78.36)
Self-reported precarious work	-28.55* (-55.26, -1.84)	-44.96* (-86.83, -3.09)	-10.96 (-43.86, 21.94)
Fixed-term employment (i.e., temporary work)	19.89 (-1.56, 41.33)	19.95 (-16.81, 56.71)	19.77 (-4.21, 43.76)

Note: Adjusted for age, marital status, education, smoking, alcohol use, self-reported health, year-fixed effect, and region-fixed effect; ∗*P* < .05. ∗∗*P* < .01 ∗∗∗*P* < .001

Table 2 presents the results of individual-level fixed-effects regression models. In Model 1, the fixed-effects model (men and women combined) for the minutes spent on LTPA per month indicated that changes in working conditions were associated with LTPA. Individual-level fixed effects models predicting the effect of changes in working conditions on participation in LTPA indicated an association between changes in working conditions and LTPA. For example, an hour increase in weekly working hours was associated with a 12.02 min decrease (95% confidence interval [CI] -19.22 to -4.83). Having labor union membership and part-time worker status were associated with 30.49 (95% CI 7.76 to 53.23) and 50.29 (95% CI 14.98 to 85.60) minutes increased LTPA. However, manual labor and precarious work conditions were associated with 42.07 (95% CI -71.86 to -12.27) and 55.26 (95% CI -55.26 to -1.84) minutes decreased LTPA.

Models 2 and 3 show the results of gender-stratified models. For both men and women, longer working hours was associated with shorter time spent on LTPA: an hour increase in weekly working hours was associated with − 0.94 (95% CI: -1.74 to -0.13) and − 1.15 (-2.07 to -0.22) minutes reductions in LTPA per month, respectively. However, union membership was associated with 44.36 min more LTPA per month (95% CI: 14.82 to 73.89) among men, whereas labor union status was not associated with a significant increase in LTPA among women. Likewise, being a part-timer was associated with 41.60 min more LTPA per month (95% CI: 3.62 to 79.58) among women, while it was not associated with a significant change among men. Additionally, while having manual labor (beta=-64.32, 95% CI: -106.36 to -22.29) and self-reported precarious worker (beta=-44.96, 95%CI: -86.83 to -3.09) were associated with reductions in time spent on LTPA among men, these associations were not observed among women.

The results of the sensitivity analyses are available in an additional File 1, Supplementary Tables 1 to Table 3. The first sensitivity results overall supported our results from the main models. The logistic fixed-effects regression models (Supplementary Table 1) showed that reductions in working hours (odds ratio [OR]: 0.99, 95% CI: 0.99 to 1.00), having a union membership (OR: 1.25, 95% CI: 1.14 to 1.38), shift work (OR: 1.18, 95% CI: 1.03 to 1.33), non-manual labor (OR: 0.80, 95% CI: 0.70 to 0.92), part-time work (OR: 1.42, 95% CI: 1.16 to 1.66), non-precarious work (OR: 0.84, 95% CI: 0.74 to 0.95), and fixed-term employment (OR: 1.11, 95% CI: 1.01 to 1.24) were associated with increased LTPA, with the effects more clearly observed in men than in women. In contrast, the analysis with the lagged outcome using both linear (Supplementary Table 2) and logit fixed effects (Supplementary Table 3) models presented changes in working conditions that appeared to have hardly varied over a short period.

## Discussion

Building upon prior studies investigating the link between employment conditions and LTPA, this research explored whether diverse employment conditions correlated with distinct LTPA levels among the working-age population in South Korea. In the gender aggregated models, labor union membership and part-time work were associated with increased LTPA, while increased working hours, manual labor, and precarious work was associated with decreased LTPA. In the gender-stratified models, both men and women exhibited a relationship between longer working hours and reduced LTPA. However, gender differences were also observed. For instance, while union membership correlated with increased LTPA in men, no significant association was found in women. Likewise, changes in LTPA participation were associated with part-time work for women, but no significant changes were observed for men. Nonetheless, in models incorporating lagged outcomes, the longitudinal association appeared to largely vanish.

Extending previous research on the cross-sectional association between employment conditions and LTPA, this study aimed to examine whether varying employment conditions were associated with differing LTPA among working-age populations in South Korea. This study focused on employment conditions, including working hours, union membership, having manual, shift, part-time,, or at-home work, having (self-reported) precarious employment conditions, or having fixed-term employment. Although the linear regression results showed that the probability of change was relatively low, ranging from 1.2 to 3.7% in terms of an increase in the probability of participation in LTPA for the gender-combined model, the logistic regression model that considered only those who had within-person changes in LTPA over the study period found much more significant effects. However, in our models that introduced lagged outcomes, the longitudinal association appeared to have mostly disappeared. We also examined whether the association between employment conditions and LTPA differed by gender. While men were clearly shown to have a longitudinal association between employment conditions and LTPA, the association was less apparent among women.

Our findings are consistent with previous research. For example, a 2015 Korean study that used KLIPS data and applied a cross-sectional design found that shorter working hours, non-manual work, and non-precarious work were associated with higher LTPA [11]. Our findings that longer working hours are associated with lower LTPA are consistent with another Korean study based on the Korea Health Panel Survey (KHPS) [21]. Although the KHPS is also a population representative survey that facilitates some comparable analyses in the present study, the KLIPS collects more comprehensive information about labor characteristics. Likewise, a study in Canada showed that working hours and significant work-related physical activity were associated with changes (from inactivity to activity) in LTPA [7]. Additionally, our study is the first to find that union membership, potentially a sign of higher job controls and satisfaction was associated with a higher likelihood of more LTPA. However, in contrast to the 2015 Korean study [11] that found that shift work was associated with a lower prevalence of LTPA, we found that a change from non-shift to shift work was associated with a higher level of LTPA. Our gender-stratified models also corroborated the findings of the Korean study, indicating that work-related factors appeared to be more associated with a higher level of LTPA among men.

In Korean society, where gender discrimination in the labor market is severe (and thus, women workers are less likely to derive incentives from work) and where intense social pressure exists for women to have a specific body shape [22], it is highly likely that women’s participation in physical activity will be more adversely affected by factors in addition to labor conditions. For example, strong traditional gender roles and expectations may dictate that women prioritize domestic duties and caregiving responsibilities which may restrict women’s opportunities and desire to engage in leisure activities, including LTPA [23, 24]. This is supported by the fact that work-family conflict is very severe in Korea [25], which may lead to time constraints and reduced energy for LTPA among women, even if they have favorable employment conditions. This could clarify why the shift from full-time employment to part-time work among women was more likely to result in increased time allocated for LTPA. Furthermore, while union membership has been found in other studies [26], to be positively associated with shortened working hours, in a 2011 study, working Korean women who reported reduced working hours often allocated extra time to housework [27, 28]. This may provide an explanation for the significant positive association between LTPA and union membership in men, but not women.

### Limitations and contributions

This study has some limitations. First, our outcome was a study-created self-report measure, which has yet to be validated. However, the KLIPS is the only available panel study in South Korea that contains data on physical activity and multiple employment conditions beyond occupation involving a nationally representative section of the population. Moreover, a panel featuring a validated measure of physical activity is also uncommon internationally [10]. Second, although our model controlled for unobserved time-invariant individual-level factors and several key time-varying variables, there may still have been unobserved confounding time-variant factors affecting the relationship between employment conditions and LTPA. Finally, we only considered LTPA and did not assess or model work-related physical activity (WPA), which may have weakened our findings, assuming that there may be offset relationships between LTPA and WPA. Nevertheless, this study contributes to the literature by providing a population-based estimate of longitudinal changes between employment conditions and LTPA derived from a nationally representative large panel dataset. In addition, our use of an individual-level fixed-effects model facilitated a more rigorous approach by allowing us to control for unobservable and observable confounders effectively.

## Conclusions

In conclusion, our study found that changes in employment conditions, including shorter working hours, having a labor union membership, a part-time job, and non-manual and non-precarious work, were associated with greater participation in LTPA in South Korean workers, especially among men. To determine relevant causal relationships, we recommend that future research use a quasi-experimental design related to an exogenous shock that brings about shifts in employment conditions. In addition, future research could explore factors influencing LTPA participation among women, given the apparent differential effects. This approach and its results would help inform public health authorities about developing more effective plans and interventions to increase LTPA participation. Specifically, this study will help to provide information on developing more effective planning and interventions, especially among those employed in manual and precarious work, to increase LTPA participation.

## Electronic supplementary material

Below is the link to the electronic supplementary material.

Supplementary Material 1

## Data Availability

The KLIPS data is publicly available through Korea Labor & Income Panel Study website. Korean Labor & Income Panel Study – Codebook (https://www.kli.re.kr/klips_eng/selectBbsNttList.do?bbsNo=73&key=261).

## Abbreviations

LTPA: Leisure-time physical activity
KLIPS: Korean Labor & Income Panel Study
WPA: Work-related physical activity
