# Supplementary material for: Employment conditions and leisure-time physical activity among Korean workers: a longitudinal study (2009–2019)
Source: BMC Public Health. 2023 May 25;23:955. doi: 10.1186/s12889-023-15766-w (PMC10214723; doi:10.1186/s12889-023-15766-w)
Supplement: Supplementary file 1 — Supplementary Material 1 [file 12889_2023_15766_MOESM1_ESM.docx]

**Supplementary Table 1. Logit results – no lagged**

| Predictors | Total | Men | Women |
| --- | --- | --- | --- |
| +1 Working hours a week | 0.99*** (0.99, 1.00) | 0.99**(0.99, 1.00) | 0.99*** (0.98, 1.00) |
| No labor union | 1 | 1 | 1 |
| Labor union | 1.25***(1.14,1.38) | 1.28***(1.13,1.43) | 1.20*(1.01,1.42) |
| Non-shift work | 1 | 1 | 1 |
| Shift work | 1.18*(1.03, 1.33) | 1.18*(1.01, 1.38) | 1.17 (0.93, 1.46) |
| Non-manual labor | 1 | 1 | 1 |
| Manual labor | 0.80**(0.70, 0.92) | 0.73***(0.62, 0.86) | 1.02 (0.80, 1.28) |
| Full-time work | 1 | 1 | 1 |
| Part-time work | 1.42***(1.16,1.66) | 1.76 (1.25, 2.46) | 1.17 (0.93, 1.46) |
| Working on-site | 1 | 1 | 1 |
| Work at home | 0.99 (0.69, 1.41) | 1.11**(0.55, 2.20) | 0.96 (0.63, 1.44) |
| Non-precarious work (self announcement) | 1 | 1 | 1 |
| Self-reported  precarious work | 0.84**(0.74, 0.95) | 0.71***(0.58, 0.84) | 1.01 (0.84, 1.20) |
| Permanent work | 1 | 1 | 1 |
| Fixed-term employment (i.e., temporary work) | 1.11*(1.01, 1.24) | 1.15 (0.98, 1.33) | 1.08 (0.93, 1.24) |

Adjusted for age, marital status, education, smoking, alcohol use, self-reported health, year-fixed effect, and region-fixed effect.

∗P < .05. ∗∗P < .01.

**Supplementary Table 2. Individual level linear fixed-effect model predicting the lagged effect of change in working and employment conditions on the time spent on leisure-time physical activity**

| Predictors | Model 1: Total | Model 2: Men | Model 3: Women |
| --- | --- | --- | --- |
| Working hours | -4.50 (-12.67, 3.68) | -2.40 (-13.92, 9.12) | -8.70 (-18.97, 1.56) |
| Labor union | 11.21 (-16.01, 38.44) | 23.05 (-13.08, 59.18) | -12.36 (-50.14, 25.43) |
| Shift work | 1.25 (-32.96, 35.46) | -6.67 (-54.40, 41.06) | 14.39 (-27.78, 56.57) |
| Manual labor | -18.89 (-49.91, 12.14) | -19.11 (-61.34, 23.13) | -11.69 (-55.29, 31.91) |
| Part-time work | 16.30 (-23.70, 56.30) | -6.46 (-95.48, 82.56) | 14.82 (-27.86, 57.50) |
| Work at home | -68.94* (-137.87, -0.01) | -69.74 (-202.62, 63.13) | -64.73 (-144.44, 14.97) |
| Self-reported  precarious work | 1.58 (-29.09, 32.26) | -17.18 (-69.02, 34.66) | 17.98 (-15.49, 51.45) |
| Fixed-term employment | 12.94 (-14.37, 40.24) | 44.00 (-2.98, 90.99) | -13.68 (-43.73, 16.37) |

Adjusted for age, marital status, education, smoking, alcohol use, self-reported health, year-fixed effect, and region-fixed effect.

∗P < .05. ∗∗P < .01.

**Supplementary Table 3. Individual level logit fixed-effect model predicting the lagged effect of change in working and employment conditions on the time spent on leisure-time physical activity**

| Predictors | Model 1: Total | Model 2: Men | Model 3: Women |
| --- | --- | --- | --- |
| +1 Working hours a week | 1.00 (0.99, 1.00) | 1.00 (0.99, 1.00) | 1.00 (0.99, 1.00) |
| No labor union | 1 | 1 | 1 |
| Labor union | 1.07 (0.96, 1.19) | 1.11 (0.97, 1.26) | 0.99 (0.82, 1.21) |
| Non-shift work | 1 | 1 | 1 |
| Shift work | 1.12 (0.97, 1.30) | 1.09 (0.91, 1.31) | 1.18 (0.90, 1.54) |
| Non-manual labor | 1 | 1 | 1 |
| Manual labor | 0.82* (0.71, 0.97) | 0.82* (0.68, 0.99) | 0.86 (0.65, 1.13) |
| Full-time work | 1 | 1 | 1 |
| Part-time work | 1.20 (0.96, 1.49) | 1.15 (0.75, 1.77) | 1.13 (0.87, 1.48) |
| Working on-site | 1 | 1 | 1 |
| Work at home | 0.71 (0.46, 1.11) | 0.73 (0.30, 1.77) | 0.74 (0.44, 1.24) |
| Non-precarious work (self announcement) | 1 | 1 | 1 |
| Self-reported  precarious work | 0.91 (0.79, 1.05) | 0.84 (0.68, 1.04) | 1.00 (0.81, 1.23) |
| Permanent work | 1 | 1 | 1 |
| Fixed-term employment (i.e., temporary work) | 1.06 (0.94, 1.20) | 1.13 (0.96, 1.35) | 1.01 (0.85, 1.19) |

Adjusted for age, marital status, education, smoking, alcohol use, self-reported health, year-fixed effect, and region-fixed effect.

∗P < .05. ∗∗P < .01.
